# Evaluation of a Free Milk in Schools Program in New Zealand: Effects on Children's Milk Consumption and Anthropometrics

**DOI:** 10.1111/josh.12649

**Published:** 2018-07-10

**Authors:** Samantha Marsh, Yannan Jiang, Karen Carter, Clare Wall

**Affiliations:** ^1^ National Institute for Health Innovation, University of Auckland, Private Bag 92019, Auckland Mail Centre, Auckland 1142, New Zealand.; ^2^ Faculty of Medical and Health Sciences, University of Auckland, 85 Park Road, Grafton, Auckland 1142, New Zealand.

**Keywords:** children, evaluation, milk, school

## Abstract

**BACKGROUND:**

While dietary sources of calcium are important for bone health, the intake of milk and milk products decreases as children get older. A free milk in schools program may mitigate this decrease in milk consumption. We evaluated the Fonterra Milk for schools program (a free milk initiative) by determining changes in children's milk consumption and anthropometric measures over a 2‐year period.

**METHODS:**

The evaluation was conducted in children aged 7‐9 years in a representative sample of primary schools participating in the free milk program, in Auckland, New Zealand. The primary outcome was the proportion of children meeting the New Zealand guidelines for dairy and milk product consumption over 7 days (≥2 servings per day). Changes in anthropometric measures were also assessed as secondary outcomes.

**RESULTS:**

Nine schools (N = 511 children) participated in the evaluation. The proportion of children meeting the New Zealand guidelines for milk and milk product consumption over 7 days increased significantly from baseline to 2 years' follow‐up (72% vs 94%; p < .001). Body mass index z scores did not change significantly between baseline and 2 years' follow‐up.

**CONCLUSIONS:**

The Fonterra Milk for Schools initiative appears to be an effective way of increasing milk/milk product consumption behaviors in school‐aged children.

Calcium and vitamin D are necessary for attainment of peak bone mass and bone health in children.^1^, ^2^ Children's diet must provide the required amount of calcium and vitamin D to satisfy the needs of the skeleton.^3^ It has been shown that children who avoid dietary calcium, in particular cow's milk, are at increased risk of small stature, poor bone health, and fractures.^3^, ^4^, ^5^, ^6^ Low consumption of dietary calcium in childhood has also been linked with an increased risk of colorectal cancer,^7^ and osteoporosis and fractures in later life.^8^, ^9^ Given the aging population, improving health through increased intake of dietary calcium in childhood may be considered a public health issue.

The primary sources of dietary calcium are milk, including full milk, low‐fat, and nonfat, and other milk products, including cheese and yoghurt.^1^ It is recommended in New Zealand^10^ and internationally^1^, ^11^ that children from the age of 1 year onwards consume milk and milk products. Specifically, New Zealand guidelines recommend 2 to 3 daily servings of milk or milk products for children.^12^, ^13^ Yet despite these recommendations, 23% of New Zealand children and young people report never drinking milk, with total milk consumption decreasing with increasing age.^14^ This decrease in consumption with increasing age is concerning. The period of adolescence, which follows childhood, is a time when milk consumption should be optimized, given that high accretion rates of calcium are necessary during this crucial period of rapid growth.^15^ It has been estimated that 26% of adult calcium is actually laid down during the adolescent years.^15^ Yet despite the increased need for calcium intake during these years, consumption of dietary sources of calcium decreases.

While adolescence represents an important period for optimizing calcium intake, it has been suggested that childhood, defined as ages 4‐8 years, is an important period for laying the foundation of dietary behaviors associated with adequate calcium intake.^1^ Therefore, efforts are needed to increase milk and milk product consumption patterns in New Zealand children. It is particularly important that these are long‐term efforts, given that supplementing calcium intake over a short time period, such as 1‐2 years, is unlikely to afford long‐term benefit, with respect to attainment and maintenance of peak bone mass.^16^ Long‐term provision of free milk to primary schools is one way of achieving this goal.

In 2011, the Milk for Schools program was introduced in New Zealand by Fonterra Co‐operative Group Limited. Fonterra is a multinational, farmer cooperative with headquarters in New Zealand. It accounts for approximately 30% of the world's dairy exports and is the largest company in New Zealand.^17^ The aim of the Milk for Schools program was to offer all primary school children access to a free 200 mL serving of reduced‐fat milk (1.5%) every day at school in order to increase milk consumption. The program, which was initially piloted in Northland, New Zealand, was extended to the Auckland region in 2013. An evaluation of the Milk for Schools program was undertaken to (1) determine changes overtime in milk consumption patterns in the cohort of children participating in the Milk for Schools program in Auckland, New Zealand, (2) assess parental perceptions of the program, and (3) investigate changes overtime in anthropometric measures.

## METHODS

In 2013 primary schools that had signed up to participate in the Milk for Schools Program but not yet commenced it were approached to take part in the study. Children enrolled in the evaluation were assessed at baseline in October/November of 2013, 1‐year follow‐up in 2014, and 2‐year follow‐up in 2015. Parental perceptions of the free milk initiative were also assessed at 1‐year follow‐up using an anonymous survey. The parent survey was not linked to the child data. Ethical approval for this study was obtained from the National Health and Disability Ethics Committee 13/NTA/155.

### Participants

Eligible participants included children in years 3 or 4, aged 7‐9 years in 2013 enrolled in an Auckland school participating in the Milk for Schools Program who were able to provide informed consent or assent to participate and could speak and understand English. Parents/caregivers aged 18 years and older were also required to be able to provide assent. For the Parental survey, eligible participants were parents of children attending schools who were taking part in the Milk for Schools evaluation.

### Procedure

Schools were stratified into New Zealand school decile quintiles ranging from 1 to 5. Decile ratings represent the socioeconomic position of the school's student community compared with other schools in New Zealand. A decile rating of 1 therefore signifies the 10% of schools with the highest proportion of students from lower socioeconomic communities.^18^ Two schools were randomly selected from each of the 5 school decile quintiles. The schools were approached by Fonterra and asked whether they would like to take part in the study. If a school declined, then another school from the same quintile was randomly selected. This process was repeated until either a school agreed to participate or no schools were left in the quintile to approach. Informed consent/assent was obtained from all children and their parents/caregivers prior to registration.

School principals were approached by Fonterra to identify those interested in participating in the research. Researchers then worked with the school to develop an appropriate recruitment process. Information sheets were provided and written informed consent to approach students was sought from principals. Invitations to participate and parent/caregiver information sheets were provided to all students of years 3 and 4. A free call number was provided for parents/caregivers to discuss the study in more detail with a member of the study team. A reminder letter and consent form were sent home on the day prior to recruitment. Parents/caregivers could refuse permission for their child to participate on the consent form provided. All students providing informed assent/consent in years 3 and 4 were given the child information sheet and consent form. Trained research assistants explained the measurements to be taken to students in a way that they could understand and also answered any questions.

### Instrumentation

All child measures were collected on‐site at each participating school. The 3 study days at baseline, year 1, and year 2 were run similarly, although written informed consent forms were only collected at the baseline visit. Participating children were assessed by the research team either in the classroom or in a space designated by the school. A pen‐and‐paper questionnaire was completed by each of the children and anthropometric data were collected. All researchers underwent a training session, during which inter‐investigator reliability was confirmed for all anthropometric measurements.

The parents of children eligible to take part in the Milk for Schools evaluation, including those who chose not to have their child involved in the free milk program, were invited to complete a survey designed to assess their attitudes about the program. Surveys were distributed by the school, with children asked to take the survey home to their parents. The survey could be completed online or using a pen‐and‐paper format at a time and location that suited them. Parents provided informed consent before completing the survey.

### Measurements

The primary endpoint was the proportion of children consuming at least 2 servings per day of milk or milk products over 7 days. This was assessed using the child pen‐and‐paper questionnaire, which assessed children's milk and milk consumption habits during a usual day before, during, and after school and during a usual weekend day. Although there are no validated questionnaires to assess milk consumption habits in this age group, the questionnaire was modified from one used previously in our research group in a similar group of children. The questionnaire was administered in a small group setting of 3‐4 children either within the classroom or in a space designated by the school. A researcher read out each item of the questionnaire and research assistants moved around the group to answer any questions and ensure the children were filling in the questionnaire correctly. All research assistants were trained in delivery of the questionnaire, and props were used to help children understand portion sizes.

Other evaluations included milk consumption on school days and weekend days, anthropometric measures, including bodyweight (kg), standing height (cm), sitting height (cm), sitting height ratio (sitting height/height × 100), and waist circumference (cm), and plain milk, flavored milk, cheese, and yoghurt consumption patterns. Each of these measures was collected at baseline, and 1‐ and 2‐year follow‐up. Bodyweight was measured to the nearest 0.1 kg, using a digital scale (Tanita, UM‐070, Arlington Heights, IL), and standing height was measured to the nearest 0.1 cm with a stadiometer (Seca, 214, Hamburg, Germany). Sitting height was measured to the nearest 0.1 cm with a stadiometer (Seca, 214) according to standard procedures,^19^ with the child sitting on the floor. Waist circumference was measured using a flexible nonelastic tape, which was placed at the level of the umbilicus. For all anthropometric assessments, 2 measurements were taken, or a third if the difference between the 2 measurements was greater than a prespecified margin for each measure, ie, >0.1 kg for bodyweight and >1 cm for height and waist circumference. The mean of 2 measurements or the median of 3 were then used for analysis.

Body mass index (BMI) was calculated using weight and height, and standardized to z score using the World Health Organization (WHO) growth standards for 5‐ to 19‐year‐olds.^20^ Participants were then classified as being underweight, normal weight, overweight, or obese.^21^, ^22^

#### *Parental survey*

The self‐report parent survey was designed to investigate parental perceptions of the milk for schools initiative, including whether there had been any perceived changes from baseline in their child's (1) milk/milk product consumption behaviors, (2) the amount of milk/milk products they offer their child at home, and (3) their child's preference for the taste of milk/milk products. Parents were given a selection of responses to choose between for each question and were asked to select one response for each question. The survey also assessed parents' perceptions about the importance of milk for childhood health, growth, and oral health. The survey was not validated before it was administered.

### Data Analysis

Individual participant data collected from all participating schools over the 2 years were recorded in a secure study database and imported to SAS version 9.4 (SAS Institute Inc. Cary, NC) for statistical analysis. Parent questionnaire survey collected at 1‐year follow‐up was summarized descriptively in frequency and percentages. Demographic information and 2‐year measures on all children followed up in the study were summarized as overall and by important subgroups. These include school quintiles, specific ethnic groups, ie, Maori, Pacific, Asian, NZ European or other, and sex. Continuous variables were presented as mean and SD. Categorical variables were presented as frequencies and percentages.

Differences in primary and secondary outcome measures at baseline, 1‐year and 2‐year follow‐up visits were next tested on those children who provided valid data at all visits. Missing data were not imputed. Both unadjusted and adjusted logistic regression analyses were conducted when all schools were included, controlling for children's ethnicity, sex and school quintiles with a random subject effect to take into account repeated measures. A p value of <5% was considered statistically significant, followed by post hoc pairwise tests between visits adjusting for multiple comparisons.

## RESULTS

Nine schools provided consent; 1 school in quintile 1, and 2 schools each in quintiles 2, 3, 4, and 5. Overall, 511 children participated in baseline data collection, 423 at 1‐year follow‐up, and 379 at 2‐year follow‐up, corresponding to a loss to follow‐up of 88 at 1 year and 132 at 2 years. Forty‐five percent of children included in the evaluation identified as New Zealand European (45%), and the sample was well‐balanced according to sex (52% female), and school year (50% year 3). No children were deemed ineligible due to language restrictions. Table 1 presents baseline demographic and anthropometric data for all students participated in the 9 schools.

**Table 1:** Descriptive Summary of Participants at Baseline (All Schools)

	Baseline (N = 511)
Demographics (mean, SD)	
Age (years)	8.5 (0.61)
Sex (n, %)	
Female	266 (52)
Male	244 (48)
Missing	1 (<1)
School year (n, %)	
Year 3/4 (2013)	253 (49.5)/255 (50)
Missing	3 (0.5)
Ethnicity (n, %)	
NZ European	229 (45)
Maori	76 (15)
Pacific	162 (32)
Chinese	13 (3)
Indian	39 (8)
Other	107 (21)
Do not know	9 (2)
Anthropometrics (mean, SD)	
Standing height (cm)	133 (7.03)
Sitting height (cm)	69 (3.43)
Weight (kg)	33 (8.68)
BMI (kg/m^2^)	18 (3.57)
zBMI (WHO)	0.94 (1.33)
Waist circumference (cm)	63 (9.24)

Significant differences in baseline anthropometric data were not shown for New Zealand European versus Asian children, except for lower zBMI scores in Asian children. Maori and Pacific children had greater measures for sitting height, weight, waist circumference, BMI, and zBMI scores (data not shown). Further, standing height and the sitting height ratio (SHR) also differed significantly between New Zealand European and Pacific children (136.38 vs 136.38 cm, p < .001; and 0.51 vs 0.52, p = .01, respectively).

Table 2 presents milk and milk product consumption data at baseline, 1 year, and 2 years, for all schools and by quintile. Significant increases over time in the proportion of children meeting the NZ guidelines for milk and milk product consumption over 7 days, during school days, and on weekends, were demonstrated over the duration of the study among all schools. The change from baseline to 2 years remained statistically significant in adjusted regression analyses controlling for multiple comparisons (p < .04). Most children (73%) reported consuming the milk provided by the Fonterra Milk for Schools program, and of those children who reported drinking the milk, the majority of them consumed the milk every day (5 days a week; 52%). Further, 99% of children reported that they usually finished the whole carton of milk, and 96% said they liked the taste of the milk provided by the program.

**Table 2:** Children Meeting NZ Milk and Milk Product Consumption Guidelines Overall and by Quintile at Baseline, and 1‐Year and 2‐Year Follow‐Up

Children Meeting NZ Milk/Milk Product Guidelines*	All Schools		Quintile 1		Quintile 2		Quintile 3		Quintile 4		Quintile 5	
	N	n (%)	N	n (%)	N	n (%)	N	n (%)	N	n (%)	N	n (%)
7 days a week												
Baseline	511	369 (72)	52	34 (65)	178	136 (76)	84	60 (71)	101	75 (74)	96	64 (67)
1 year	423	320 (76)	34	22 (65)	144	105 (73)	69	54 (78)	90	75 (83)	86	64 (74)
2 years	379	318 (84)	40	30 (75)	127	114 (90)	61	50 (82)	82	66 (81)	69	58 (84)
#p value		p = .0003		n.s.		p = .0028		n.s.		n.s.		n.s.
Adjusted p value		p = .0002										
On week days												
Baseline	511	432 (85)	52	43 (83)	178	155 (87)	84	70 (83)	101	87 (86)	96	77 (80)
1 year	423	376 (89)	34	31 (91)	144	129 (90)	69	62 (90)	90	82 (91)	86	72 (84)
2 years	379	354 (93)	40	37 (93)	127	122 (96)	61	56 (92)	82	76 (93)	69	63 (91)
p value		p = .0003		n.s.		p = .0423		n.s.		n.s.		n.s.
Adjusted p value		p = .0003										
On weekends												
Baseline	511	402 (79)	52	36 (69)	178	146 (82)	84	64 (76)	101	80 (79)	96	76 (79)
1 year	423	340 (80)	34	25 (74)	144	112 (78)	69	57 (83)	90	76 (84)	86	70 (81)
2 years	379	323 (85)	40	30 (75)	127	116 (91)	61	50 (82)	82	67 (82)	69	60 (87)
p value		p = .0442		n.s.		p = .0135		n.s.		n.s.		n.s.
Adjusted p value		p = .0406										

# Unadjusted and adjusted logistic regressions were conducted to test the change in proportion of children meeting NZ guidelines over time.

* At least 2 servings per day of milk and/or milk products.

While anthropometric measures increased significantly from baseline overall, no significant changes in zBMI scores were observed overall (all schools) or for the individual quintiles (Table 3). Further, the proportion of children within each BMI category did not differ significantly from baseline when assessed overall (all schools), or by ethnic group or sex (Table 4).

**Table 3:** Comparative Children's Anthropometric Data by Quintile at Baseline, and 1‐Year and 2‐Year Follow‐Up

Anthropometrics (Mean, SD)	All schools	Quintile 1	Quintile 2	Quintile 3	Quintile 4	Quintile 5
Standing height						
Baseline	133 ± 7.0 cm	137 ± 7.0 cm	133 ± 7.2 cm	133 ± 7.4 cm	133 ± 6.5 cm	132 ± 6.6 cm
1 year	139 ± 7.5 cm	143 ± 7.2 cm	140 ± 7.5 cm	139 ± 8.0 cm	139 ± 7.2 cm	138 ± 7.1 cm
2 years	145 ± 8.0	149 ± 8.3	145 ± 7.9	145 ± 8.5	144 ± 7.5	144 ± 7.4
p value	p < .0001	P < .0001	p < .0001	p < .0001	p < .0001	p < .0001
*p value	p < .0001					
Sitting height						
Baseline	69 ± 3.4 cm	70 ± 3.7 cm	69 ± 3.6 cm	69 ± 3.7 cm	69 ± 3.0 cm	68 ± 3.1 cm
1 year	71 ± 3.5 cm	73 ± 3.9 cm	71 ± 3.4 cm	71 ± 3.8 cm	71 ± 3.4 cm	71 ± 3.3 cm
2 years	73 ± 3.9	75 ± 4.4	73 ± 3.8	73 ± 4.4	73 ± 3.6	73 ± 3.6
p value	p < .0001	p < .0001	p < .0001	p < .0001	p < .0001	p < .0001
*p value	p < .0001					
Sitting height ratio						
Baseline	51.5 ± 1.4	51.0 ± 1.4	51.4 ± 1.4	51.6 ± 1.5	51.7 ± 1.5	51.8 ± 1.3
1 year	51.1 ± 1.4	50.9 ± 1.3	51.0 ± 1.4	51.0 ± 1.4	51.3 ± 1.4	51.4 ± 1.3
2 years	50.5 ± 1.4	50.3 ± 1.4	50.4 ± 1.4	50.3 ± 1.6	50.7 ± 1.2	50.7 ± 1.3
p value	p < .0001	n.s	p < .0001	p < .0001	p < .0001	p < .0001
*p value	p < .0001					
Weight						
Baseline	33 ± 8.7 kg	39 ± 11.5 kg	34 ± 8.7 kg	32 ± 8.4 kg	32 ± 7.5 kg	30 ± 6.1 kg
1 year	38 ± 10.6 kg	47 ± 15.1 kg	39 ± 10.6 kg	36 ± 9.0 kg	36 ± 9.7 kg	34 ± 7.7 kg
2 years	44 ± 13.0	56 ± 18.2	45 ± 12.0	42 ± 12.6	40 ± 10.3	39 ± 9.5
p value	p < .0001	p < .0001	p < .001	p < .0001	p < .0001	p < .0001
*p value	p < .0001					
BMI						
Baseline	18 ± 3.6 kg/m^2^	21 ± 4.8 kg/m^2^	19 ± 3.7 kg/m^2^	17 ± 3.2 kg/m^2^	18 ± 3.0 kg/m^2^	17 ± 2.3 kg/m^2^
1 year	19 ± 4.2 kg/m^2^	23 ± 5.9 kg/m^2^	20 ± 4.4 kg/m^2^	19 ± 3.5 kg/m^2^	19 ± 3.6 kg/m^2^	18 ± 2.7 kg/m^2^
2 years	20 ± 4.8 kg/m^2^	25 ± 6.6	21 ± 4.8	20 ± 4.4	19 ± 3.6	19 ± 3.2
p value	p < .0001	p = .0081	p < .0001	p = .0128	p = .0243	p = .0008
*p value	p < .0001					
zBMI (WHO):						
Baseline	0.95 ± 1.33	1.70 ± 1.42	1.23 ± 1.40	0.62 ± 1.32	0.79 ± 1.20	0.51 ± 1.00
1 year	0.99 ± 1.32	1.92 ± 1.42	1.25 ± 1.43	0.71 ± 1.28	0.79 ± 1.16	0.61 ± 1.03
2 years	1.09 ± 1.34	2.03 ± 1.49	1.37 ± 1.34	0.74 ± 1.37	0.82 ± 1.09	0.64 ± 1.11
p value	n.s	n.s.	n.s.	n.s.	n.s.	n.s.
*p value	n.s.					
Waist circumference						
Baseline	63 ± 9.2 cm	68 ± 10.3 cm	65 ± 10.2 cm	62 ± 8.9 cm	62 ± 8.2 cm	59 ± 5.7 cm
1 year	65 ± 10.0 cm	72 ± 11.7 cm	67 ± 10.7 cm	64 ± 8.6 cm	64 ± 9.3 cm	62 ± 7.9 cm
2 years	69 ± 10.7	77 ± 13.7	71 ± 10.9	67 ± 9.3	66 ± 8.6	65 ± 7.9
p value	p < .0001	p < .0001	p < .0001	p = .0011	p = .0434	p < .0001
*p value	p < .0001					

n.s., nonsignificant.

p value = Repeated measures analysis to test any difference in outcomes among 3 visits, as overall and by quintile.

* p value = Repeated measures analysis test on all schools to any difference in outcomes among 3 visits, adjusted for school quintiles, ethnic groups and sex.

**Table 4:** BMI Categories at Baseline, and 1‐Year and 2‐Year Follow‐Up (All Schools, and By Ethnic Group and Sex)

BMI Categories (n, %)	Baseline (N = 511)	1‐Year Follow‐Up (N = 423)	2‐Year Follow‐Up (N = 379)	Difference Among 3 Visits p Value*
*All schools*				.6942
Underweight	16 (3.1)	9 (2.1)	7 (1.9)	
Healthy weight	307 (60.1)	253 (59.8)	218 (57.5)	
Overweight	109 (21.3)	100 (23.6)	88 (23.2)	
Obese	76 (14.9)	60 (14.2)	65 (17.2)	
Missing	3 (0.6)	1 (0.2)	1 (0.2)	
*Ethnic group*				
Maori:				.8525
Underweight	2 (2.6)	1 (1.7)	2 (3.6)	
Healthy weight	39 (51.3)	28 (46.7)	25 (45.5)	
Overweight	19 (25.0)	21 (35.0)	19 (34.6)	
Obese	16 (21.1)	10 (16.7)	9 (16.4)	
Missing	0	0	0	
Pacific				.8383
Underweight	0	0	0	
Healthy weight	44 (37.9)	30 (34.5)	28 (31.8)	
Overweight	28 (24.1)	25 (28.7)	23 (26.1)	
Obese	42 (36.2)	32 (36.8)	37 (42.1)	
Missing	2 (1.7)	0	0	
Asian				.9290
Underweight	6 (13.0)	4 (10.5)	2 (6.5)	
Healthy weight	29 (63.0)	26 (68.4)	20 (64.5)	
Overweight	10 (21.7)	7 (18.4)	7 (22.6)	
Obese	1 (2.2)	1 (2.6)	2 (6.5)	
Missing	0	0	0	
NZEO				.8957
Underweight	8 (2.9)	4 (1.7)	3 (1.5)	
Healthy weight	195 (71.4)	169 (71.0)	145 (70.7)	
Overweight	52 (19.1)	47 (19.8)	39 (19.0)	
Obese	17 (6.2)	17 (7.1)	17 (8.3)	
Missing	1 (0.4)	1 (0.4)	1 (0.5)	
*Sex*				
Boys				.5326
Underweight	8 (3.3)	4 (1.9)	2 (1.1)	
Healthy weight	153 (62.7)	135 (65.5)	110 (61.1)	
Overweight	52 (21.3)	38 (18.5)	35 (19.4)	
Obese	31 (12.7)	28 (13.6)	32 (17.8)	
Missing	0	1 (0.5)	1 (0.6)	
Girls				.7263
Underweight	8 (3.0)	5 (2.3)	5 (2.5)	
Healthy weight	154 (57.9)	118 (54.4)	108 (54.3)	
Overweight	57 (21.4)	62 (28.6)	53 (26.6)	
Obese	45 (16.9)	32 (14.8)	33 (16.6)	
Missing	2 (0.8)	0	0	

Underweight = BMI <18.5 kg/m^2^; Healthy weight = BMI 18.5‐24.9 kg/m^2^; Overweight = BMI 25.0‐29.9 kg/m^2^; Obese = BMI ≤30 kg/m^2^.

* Chi‐square test or Fischer's exact test was used to test any difference in BMI categories among 3 visits, as overall and by subgroups.

A total of 141 parents completed the parent questionnaire, of which 121 reported that they had signed their child up for the program. Results from the questionnaire are presented in Table 5.

**Table 5:** Parent Perceptions of Milk

	Parents (N = 141)
Parental attitudes and behaviors (n, %):	
Program has positively impacted child's health	102 (72)
Program has increased child's taste preference for milk/milk products	67 (48)
Agree that milk is important for child's:	
Health	135 (96)
Growth	135 (96)
Teeth	130 (92)
More likely to offer child milk at home	37 (26)

## DISCUSSION

The current evaluation was designed to investigate the impact of the Milk for Schools initiative on milk/milk product consumption in primary school children in Auckland, New Zealand. Findings from the evaluation suggest that the proportion of children meeting New Zealand guidelines for milk/milk product consumption significantly increased from baseline, before commencing the program, in the cohort of children participating in the milk for schools initiative. Further, parents of children participating in the initiative perceived the milk program to be very successful, reporting that it had a positive impact on their child's health and their child's preference for the taste of milk and milk products.

The evaluation provides an in‐depth look at consumption patterns of milk/milk products in a representative sample of New Zealand primary schools over a period of 2 years. Although the initial recruitment target was 300 participants, 511 participants provided consent and took part in the evaluation, with high participant retention over the 2‐year evaluation period (74%), suggesting that recruiting participants through schools may be an effective strategy. A standardized protocol for data collection was developed, which involved initial training, in addition to once‐yearly refresher training sessions, for all researchers involved in the data collection process, in order to reduce interobserver variability.

### Limitations

The evaluation was subject to a number of study limitations. First, there was no comparator group. Further, both the main study and the parent survey were subject to sampling bias, given that the schools/parents could opt in or out of the study/survey and therefore there may have been inherent differences in those who chose to participate compared with those who chose not to. With respect to the schools included in the evaluation, every effort was made to ensure a representative sample was obtained; however, we were only able to recruit one school in quintile 1, and therefore quintile 1 schools were underrepresented in our sample. There was also a high risk for recall bias, given that young children were asked to recall their milk/milk product consumption patterns. Further, although the same questionnaire was used at baseline and 1‐ and 2‐year follow‐up, changes over time in children's comprehension of the questionnaire and ability to accurately recall their consumption patterns were likely to have changed substantially overtime, and as such may have impacted the study results. However, the primary findings from the child questionnaire regarding milk/milk product consumption were supported by findings from the parental survey. Another study limitation was that the parental survey relied on self‐report and as such may have resulted in parents providing socially desirable responses to questions regarding the free milk program and their child's milk consumption behaviors. Validity and reliability measures for the survey items were also unknown, although the child questionnaire had been pretested in a previous study conducted in by researchers in our department. Finally, the evaluation only sought to gather information from the children and parents; however, information from teachers and administration staff at the school may also have been useful in establishing acceptance of the Milk for Schools program.

### Comparison with Other Studies

The primary finding, that the proportion of children in participating schools meeting the New Zealand guidelines for milk/milk product consumption increased significantly from baseline, is in contrast to national findings, which show that milk consumption patterns decrease as children get older.^14^ Although we cannot say with any certainty that the milk initiative resulted in an increase in milk/milk product consumption (given the lack of control group), the results from this evaluation, when compared with national findings, suggest that the Milk for Schools initiative appears to attenuate reductions in milk consumption in children over time. Further, our findings are in line with a 2013 evaluation of a School Milk Scheme (SMS) in Europe.^23^ According to the external evaluation, the SMS was associated with an increase in children's milk consumption, and provided a positive move towards a long‐term increase in the consumption of milk and milk products. Unfortunately, although a number of other studies have evaluated the effects of free school milk initiatives in the past, these studies have not reported the effects of the initiative on milk consumption behaviors, but instead on changes in height.^24^, ^25^

We propose 2 potential mechanisms by which the Milk for Schools initiative increased the proportion of children meeting the milk/milk product consumption guidelines on school days in participating schools. First, given that the New Zealand guidelines recommend at least 2 servings per day of milk/milk products, offering children an additional serving at school, ie, half the daily recommendation, is likely to have increased the number of children meeting guidelines. Another potential explanation for the change in milk/milk product consumption may be that taste preferences changed, via either repeat exposure or social factors. Indeed, 48% of parents reported that the program had increased their child's taste preference for milk. With respect to repeat exposure, it has been shown that learned taste acceptance, that is a change in preference for a food, can be brought about by repeated exposures to that food.^26^, ^27^ Given that children in participating schools are offered or exposed to milk every school day, repeat exposure may play a role in changing their taste preferences for milk and milk products. Similarly, social factors have been shown to shape children's preferences for food, with children's consumption of disliked foods increasing when they observe peers selecting and eating the disliked food.^28^ A school‐based program is perhaps an ideal environment for setting up opportunities for children to observe their peers consuming a food/beverage that they themselves may previously have had a dislike for. Indeed, both the WHO^29^ and Centers for Disease Control and Prevention^30^ support school‐based programs as a means for promoting lifelong healthy eating. Finally, over a quarter of parents reported that since the introduction of the Milk for Schools program, they now were more likely to offer their child milk at home, which in turn may have increased the proportion of children meeting national guidelines for milk/milk product consumption.

It has been proposed that in order to establish good bone health, children require a balanced diet of low‐fat milk and milk products, in addition to fruit and vegetables and adequate physical activity^1^; however, given the current epidemic of childhood obesity, it is necessary to assess whether a free milk in schools program has a negative effect on BMI. Indeed, while school milk initiatives in the 1930s were introduced to address malnutrition and concerns about growth and development,^24^, ^31^, ^32^ environmental changes since this time mean that different issues face children today. According to 2013 data, the worldwide prevalence of overweight and obesity in children and adolescents in developed countries is now 23.8% and 22.6% in boys and girls, respectively.^33^ New Zealand is not exempt, with the rate of obesity in children aged 2‐14 years increasing significantly between 2006/2007 and 2012/2013.^34^ Although consumption of milk in adults has been shown to be inversely related with BMI,^35^ the effect of milk in children and adolescents is less clear.^36^ One small longitudinal study in preschool children (N = 53) found that increased calcium intake and greater number of dairy servings were associated with lower body fat.^37^ Similarly, an inverse relationship between frequency of milk consumption and body mass was also demonstrated in a cross‐sectional study of 1087 children (mean age 7.5 years),^38^ and in adolescent girls (N = 323).^39^ Further, low intake of dairy in preschool children has also been shown to be associated with greater increases in body fat throughout childhood.^40^ However, findings of the protective effect of milk on childhood overweight are conflicting, with one longitudinal study in 9‐ to 14‐year‐olds (N = 12,829) suggesting that those who drank more milk were at greater risk for gaining more weight.^41^ Our evaluation of the Milk for Schools program found that BMI z scores did not change significantly from baseline after 2 years, and as such, are in line with previous research which has demonstrated a null effect of milk and dairy consumption on bodyweight in children.^42^, ^43^

### Conclusions

Overall, findings from the Milk for Schools evaluation provide a useful and timely contribution to the research regarding the ability of an in‐school initiative to increase the proportion of children meeting recommended dietary guidelines. Based on the findings from this evaluation, we conclude that the Milk for Schools initiative appears to be an effective way of increasing milk/milk product consumption behaviors in school‐aged children, without increasing BMI z scores, while also improving taste preferences and parental behaviors in the home.

## IMPLICATIONS FOR SCHOOL HEALTH

The findings from this evaluation of the Milk for Schools program demonstrate the role schools can play in delivering initiatives that help children meet important daily dietary targets. Specifically, our research shows that the school environment presents an opportunity to mitigate longitudinal changes in milk intake. Milk is an important source of nutrients for children; providing free unflavored milk with no added sugar to school children could prevent the decline in milk consumption seen at this age, and in turn the decline in nutrient intake.

Both promoting and providing healthy food in schools has been shown to improve food choices and may have an influence on long‐term food behavior and health. This ability to influence food choice is known as choice architecture and has been used in a number of school settings to influence healthy food behaviors, such as increasing the consumption of plant based foods.^44^

There are 2 main mechanisms that may have led to the increased consumption of milk in the school setting in this study: (1) increased availability of milk, which incurred no extra cost or resource burden to the school, and (2) changes overtime in taste preferences due to repeated exposure and social modeling. This study was conducted in primary school aged children and demonstrated increase in milk intake over a 2‐year period; however, it is not known yet if this strategy will ensure sustained intake into adolescence.

### Human Subjects Approval Statement

Ethical approval for this study was obtained from the National Health and Disability Ethics Committee (13/NTA/155).

## References

[1] Greer FR , Krebs NF . Optimizing bone health and calcium intakes of infants, children, and adolescents. Pediatrics. 2006;117(2):578‐585. 16452385 10.1542/peds.2005-2822

[2] McGuire S . US Department of Agriculture and US Department of Health and Human Services, dietary guidelines for Americans, 2010. Washington, DC: US Government Printing Office, January 2011. Adv Nutr. 2011;2(3):293‐294. 22332062 10.3945/an.111.000430 PMC3090168

[3] Black RE , Williams SM , Jones IE , Goulding A . Children who avoid drinking cow milk have low dietary calcium intakes and poor bone health. Am J Clin Nutr. 2002;76(3):675‐680. 12198017 10.1093/ajcn/76.3.675

[4] Wiley AS . Consumption of milk, but not other dairy products, is associated with height among US preschool children in NHANES 1999‐2002. Ann Hum Biol. 2009;36(2):125‐138. 19241191 10.1080/03014460802680466

[5] Goulding A , Cannan R , Williams S , Gold E , Taylor R , Lewis‐Barned N . Bone mineral density in girls with forearm fractures. J Bone Miner Res. 1998;13(1):143‐148. 9443800 10.1359/jbmr.1998.13.1.143

[6] Goulding A , Jones I , Taylor R , Manning P , Williams S . More broken bones: a 4‐year double cohort study of young girls with and without distal forearm fractures. J Bone Miner Res. 2000;15(10):2011‐2018. 11028455 10.1359/jbmr.2000.15.10.2011

[7] Cox B , Sneyd MJ . School milk and risk of colorectal cancer: a national case‐control study. Am J Epidemiol. 2011;173(4):394‐403. 21228415 10.1093/aje/kwq390

[8] Panel NIHC . NIH consensus conference. Optimal calcium intake. NIH consensus development panel on optimal calcium intake. JAMA. 1994;272:1942‐1948. 7990248

[9] Birnie K , Ben‐Shlomo Y , Gunnell D , et al. Childhood milk consumption is associated with better physical performance in old age. Age Ageing. 2012;41(6):776‐784. 22542496 10.1093/ageing/afs052 PMC3476828

[10] Ministry of Health . Food and Nutrition Guidelines for Healthy Infants and Toddlers (Aged 0–2): A Background Paper – Partially revised December 2012. Wellington; 2008.

[11] Dietary Guidelines Advisory Committee. Dietary guidelines for Americans 2005. Washington, DC: US Government Printing Office; 2005.

[12] Ministry of Health . Food and Nutrition Guidelines for Healthy Children Aged 2‐12 Years: A Background Paper. Wellington: Ministry of Health; 1997.

[13] Ministry of Health . Food and Nutrition Guidelines for Healthy Adolescents: A Background Paper. Wellington: Ministry of Health; 1998.

[14] Clinical Trials Research Unit . Synovate. A National Survey of Children and Young People's Physical Activity and Dietary Behaviours in New Zealand: 2008/09 – Key Findings. Wellington: Ministry of Health; 2010.

[15] Bailey DA , Martin AD , McKay HA , Whiting S , Mirwald R . Calcium accretion in girls and boys during puberty: a longitudinal analysis. J Bone Miner Res. 2000;15(11):2245‐2250. 11092406 10.1359/jbmr.2000.15.11.2245

[16] Wosje KS , Specker BL . Role of calcium in bone health during childhood. Nutr Rev. 2000;58(9):253‐268. 11060996 10.1111/j.1753-4887.2000.tb01879.x

[17] National Business Review . NZX launches milkpowder futures. *National Business Review* 4 June 2009; 2011.

[18] Ministry of Education . School deciles. Available at: http://www.education.govt.nz/school/running-a-school/resourcing/operational-funding/school-decile-ratings. Accessed July 20, 2016.

[19] Lohman TG , Roche AF , Martorell R . Anthropometric Standardization Reference Manual; Medicine & Science in Sports & Exercise 1992;24:952.

[20] Md O , Onyango AW , Borghi E , Siyam A , Nishida C , Siekmann J . Development of a WHO growth reference for school‐aged children and adolescents. Bull World Health Organ. 2007;85(9):660‐667. 18026621 10.2471/BLT.07.043497 PMC2636412

[21] Cole TJ , Bellizzi MC , Flegal KM , Dietz WH . Establishing a standard definition for child overweight and obesity worldwide: international survey. BMJ. 2000;320(7244):1240‐1243. 10797032 10.1136/bmj.320.7244.1240 PMC27365

[22] Cole TJ , Flegal KM , Nicholls D , Jackson AA . Body mass index cut offs to define thinness in children and adolescents: international survey. BMJ. 2007;335(7612):194. 17591624 10.1136/bmj.39238.399444.55 PMC1934447

[23] Directorate General for Agriculture and Rural Development . Report on the Results of the Evaluation of the School Fruit and Vegetables and School Milk Schemes against the Principles of Subsidiarity, Proportionality and Better Regulation. Brussels: European Commission; 2015.

[24] Baker IA , Elwood PC , Hughes J , Jones M , Moore F , Sweetnam PM . A randomised controlled trial of the effect of the provision of free school milk on the growth of children. J Epidemiol Community Health. 1980;34(1):31‐34. 6892711 10.1136/jech.34.1.31 PMC1052036

[25] Rona RJ , Chinn S . School meals, school milk and height of primary school children in England and Scotland in the eighties. J Epidemiol Community Health. 1989;43(1):66‐71. 2592893 10.1136/jech.43.1.66 PMC1052793

[26] Birch LL , Marlin DW . I don't like it; I never tried it: effects of exposure on two‐year‐old children's food preferences. Appetite. 1982;3(4):353‐360. 7168567 10.1016/s0195-6663(82)80053-6

[27] Pliner P . The effects of mere exposure on liking for edible substances. Appetite. 1982;3(3):283‐290. 7159080 10.1016/s0195-6663(82)80026-3

[28] Birch LL . Development of food preferences. Annu Rev Nutr. 1999;19(1):41‐62. 10448516 10.1146/annurev.nutr.19.1.41

[29] World Health Organization . Nutrition‐Friendly Schools Initiative (NFSI); 2012 [WWW document]. URL http://www.who.int/nutrition/topics/nutrition_friendly_schools_initiative/en/index.html (accessed 20 February 2017).

[30] Centers for Disease Control and Prevention . Guidelines for school health programs to promote lifelong healthy eating. J Sch Health. 1997;67(1):0‐9. 10.1111/j.1746-1561.1997.tb06289.x 8990041

[31] Ministry for Culture and Heritage . End of Free School Milk. 2001; Available at: http://www.nzhistory.net.nz/end-of-free-school-milk. Accessed July 16, 2014.

[32] Atkins PJ . Fattening children or fattening farmers? School milk in Britain, 1921–19411. Econ Hist Rev. 2005;58(1):57‐78.

[33] Ng M , Fleming T , Robinson M , et al. Global, regional, and national prevalence of overweight and obesity in children and adults during 1980–2013: a systematic analysis for the Global Burden of Disease Study 2013. Lancet. 2014;384:766‐781. 24880830 10.1016/S0140-6736(14)60460-8 PMC4624264

[34] Ministry of Health . New Zealand Health Survey: annual update of key findings 2012/13. Wellington: Ministry of Health; 2013.

[35] Zemel MB . Role of calcium and dairy products in energy partitioning and weight management. Am J Clin Nutr. 2004;79(5):907S‐912S. 15113738 10.1093/ajcn/79.5.907S

[36] Huang TTK , McCrory MA . Dairy intake, obesity, and metabolic health in children and adolescents: knowledge and gaps. Nutr Rev. 2005;63(3):71‐80. 15825809 10.1111/j.1753-4887.2005.tb00124.x

[37] Carruth B , Skinner J . The role of dietary calcium and other nutrients in moderating body fat in preschool children. Int J Obes. 2001;25(4):559‐566. 10.1038/sj.ijo.0801562 11319662

[38] Barba G , Troiano E , Russo P , Venezia A , Siani A . Inverse association between body mass and frequency of milk consumption in children. Br J Nutr. 2005;93(01):15‐19. 15705220 10.1079/bjn20041300

[39] Novotny R , Daida YG , Acharya S , Grove JS , Vogt TM . Dairy intake is associated with lower body fat and soda intake with greater weight in adolescent girls. J Nutr. 2004;134(8):1905‐1909. 15284374 10.1093/jn/134.8.1905

[40] Moore LL , Bradlee ML , Gao D , Singer MR . Low dairy intake in early childhood predicts excess body fat gain. Obesity. 2006;14(6):1010‐1018. 16861606 10.1038/oby.2006.116

[41] Berkey CS , Rockett HR , Willett WC , Colditz GA . Milk, dairy fat, dietary calcium, and weight gain: a longitudinal study of adolescents. Arch Pediatr Adolesc Med. 2005;159(6):543‐550. 15939853 10.1001/archpedi.159.6.543

[42] Newby P , Peterson KE , Berkey CS , Leppert J , Willett WC , Colditz GA . Beverage consumption is not associated with changes in weight and body mass index among low‐income preschool children in North Dakota. J Am Diet Assoc. 2004;104(7):1086‐1094. 15215766 10.1016/j.jada.2004.04.020

[43] Fiorito LM , Marini M , Francis LA , Smiciklas‐Wright H , Birch LL . Beverage intake of girls at age 5 y predicts adiposity and weight status in childhood and adolescence. Am J Clin Nutr. 2009;90(4):935‐942. 19692492 10.3945/ajcn.2009.27623 PMC2744622

[44] Ensaff H , Homer M , Sahota P , Braybrook D , Coan S , McLeod H . Food choice architecture: an intervention in a secondary school and its impact on students' plant‐based food choices. Nutrients. 2015;7(6):4426‐4437. 26043039 10.3390/nu7064426 PMC4488793

